# Commentary: Hidradenitis Suppurativa: A Systematic Review Integrating Inflammatory Pathways Into a Cohesive Pathogenic Model

**DOI:** 10.3389/fimmu.2019.00302

**Published:** 2019-02-25

**Authors:** John W. Frew

**Affiliations:** Laboratory of Investigative Dermatology, The Rockefeller University, New York, NY, United States

**Keywords:** hidradenitis suppurativa, hidradenitis, pathogenesis and diagnosis, cytokines, microbiome, genetics

We read with great interest the article “*Hidradenitis Suppurativa: A Systematic Review Integrating Inflammatory Pathways Into a Cohesive Pathogenic Model”* by Vossen et al. (1). The authors have admirably integrated data from genetic, cytokine, and microbiological studies in Hidradenitis Suppurativa (HS) into a three-phase pathogenic model of disease.

However, we have concerns that inherent bias in the collated data may introduce inaccuracies into the proposed model, and that highlighting the “*known unknowns”* in the pathogenesis of HS is needed to advance our knowledge of this disease.

## Critical Evaluation of Genetic Polymorphisms in HS using ACGM Criteria

Evidence for the role of gamma secretase sequence variants and notch signaling in HS is strong but incomplete (2). Critical evaluation of known sequence variants (2) has highlighted a “*likely pathogenic”* role for two of the three variants reported by Vossen et al. (1) (c.223G>A, c.582+1delG) and “*uncertain significance”* to the remaining variant (c.647A>C) as defined by American College of Genetic Medicine (ACGM) criteria (2). A recent systematic review (2) identified 17 of 41 variants in HS as of “*uncertain significance.”* Genome Wide Association Studies (GWAS) as well as functional and proteomic data linking identified sequence variants to the inflammatory mechanisms driving HS are currently not available. Both areas of research are vital in further evaluating the role of genetic variants in HS pathogenesis. It remains plausible that a complex polygenic model better describes observations in some cases of familial HS (2). This further emphasizes the importance of undertaking GWAS in this disorder.

## Differences in Notch signaling, the Th17 axis and Epidermal Differentiation in Human and Murine Models

Vossen et al. (1) correctly remark that the “*many substrates*” (1) of gamma secretase imply that Notch signaling may not be the sole causative pathway resulting in active disease in HS (2). In light of this, one must heed caution in extrapolating the results of gamma secretase knockout mouse models to human disease, particularly given the known differences in Notch-AhR-IL-22-Th17 pathways between mice and humans (3, 4). This is especially pertinent given the role of the Th17 axis in HS (1, 5). Murine models of AhR stimulation demonstrate a concomitant elevation of IL-22 and IL-17A production whereas human IL-22 production is accompanied by IL-17A reduction due to expansion of IL22+ IL-17- CD4+ T cells (4). This additional layer of complexity may explain discrepancies in studies of lesional IL-17 levels in HS, but also highlights the caution required in interpreting immunological data from animal models in HS (6). Additionally, gamma-secretase-Notch pathway knockout mouse models demonstrate disturbed epidermal differentiation (both delayed and premature spinous differentiation), barrier function (leading to lethal phenotypes) and alopecia (7). Such manifestations are not seen in human cases of loss-of-function variants in the gamma-secretase-Notch pathway (2, 5, 6) implying the possibility of important differences in the roles of the gamma-secretase-Notch pathway in epidermal differentiation between murine and human models as previously discussed by van der Zee et al. (6).

## Mechanical Friction and Body-Fold Occlusion May Indirectly Drive HS via Increasing Localized Moisture and pH Encouraging Proteolytic Activity of *Porphyromonas* sp.

The follicular occlusion paradigm and the role of mechanical friction has been a long-standing component of HS pathophysiology (1). However, in other diseases such as acne, evidence is emerging that microcomedone formation may be secondary to inflammation (8) (mediated by IL-1α) bringing into question whether follicular occlusion is a primary or secondary phenomenon in HS (9). The role of occlusion and friction can be re-examined in light of insights into the microbiological contribution to HS pathogenesis. Obesity, heat and occlusion all have direct alterations to the cutaneous microbiome through elevation in pH and increases in moisture (10), both conditions which favor the proteolytic activity of implicated bacteria including *porphyromonas* sp. (10, 11), as well as the aberrant innate immune response to such bacteria hypothesized to be an inflammatory driver in select cases of HS (9, 11).

## Treatment, Selection, and Analytical Bias in HS Cytokine Studies

In examining the evidence from studies regarding inflammatory pathways in HS, many published studies suffer from treatment, selection, and analytical bias (12). The studies reporting alterations in serum IL-1β, IL-6, IL-8, IL-10, IL-12p70, TNF-α, and IL-17A were conducted on patients undergoing active treatment (including Adalimumab), whilst measurement of lesional IL-32 and IL-36, TNF-α and IL-17A was conducted 3–8 weeks after withdrawal of active treatment (11). Variations in the ratio of disease severity (measured by Hurley staging), BMI and proportion of smokers may also influence the results of these studies. Also, differences in analytical techniques (ELISA vs. electrochemiluminescence) may influence the accuracy of data (11). Future studies need to control or stratify for these potential confounders, or at a minimum acknowledge the potential influence upon results.

## The Need for Further Mechanistic Studies in HS

Our understanding of the pathogenesis of HS is far from complete. Critical evaluation of mechanistic studies in HS is necessary in order to compile an accurate, reproducible model of disease. Identifying the major areas in which knowledge is deficient or mechanisms unclear is the only method or rectifying these areas of deficiency. For example, a major unknown in the pathogenesis of HS are the mechanism(s) underlying the development of sinus tracts. Vossen et al. (1) discuss the roles of proliferating epithelial strands, matrix metalloproteinases and TGF-B, however the exact mechanisms remain unclear. It is also unknown why some patients develop aggressive tract and scar formation and others do not (1, 9). The histologic features of early tract formation in HS are reminiscent of impaired wound healing and epithelial-mesenchyme transition (demonstrated by levels of TGF-β, MMP2, and ICAM-1) (9). Investigating these molecular mechanisms may help in the identification of patient at risk for aggressive tract formation or identify new therapies to prevent or ameliorate existing disease.

Vossen et al. (1) are to be commended for their efforts in synthesis of their pathogenic model for HS, however we wish to highlight the “*known unknowns*” in our understanding of this disease and the impact that bias in existing data may have in our current understanding of HS pathogenesis.

## Author Contributions

JF is responsible for the conception, design, writing, and review of this manuscript.

### Conflict of Interest Statement

The author declares that the research was conducted in the absence of any commercial or financial relationships that could be construed as a potential conflict of interest.
